# Supplementary material for: A method for high‐throughput production of sequence‐verified DNA libraries and strain collections
Source: Mol Syst Biol. 2017 Feb 13;13(2):913. doi: 10.15252/msb.20167233 (PMC5327727; doi:10.15252/msb.20167233)
Supplement: Supplementary file 1 — Expanded View Figures PDF [file MSB-13-913-s001.pdf]

Expanded View Figures

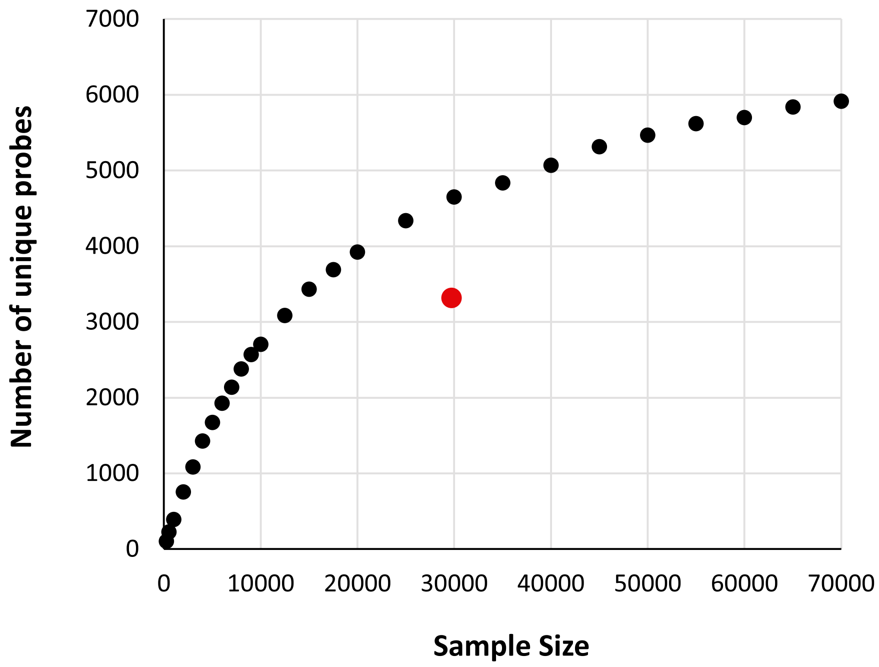

Figure EV1. Simulating the rate of recovering perfect DNA from an array-synthesized oligo pool.
Computational random sampling of the DNA sequences amplified from the array-synthesized molecular probe library. The number of unique, sequence-perfect probes is plotted on the *y*-axis, as a function of sample size on the *x*-axis. The red point represents the 3,316 sequence-verified DNA probes that were experimentally identified following screening of 29,750 *MAT*α transformants.

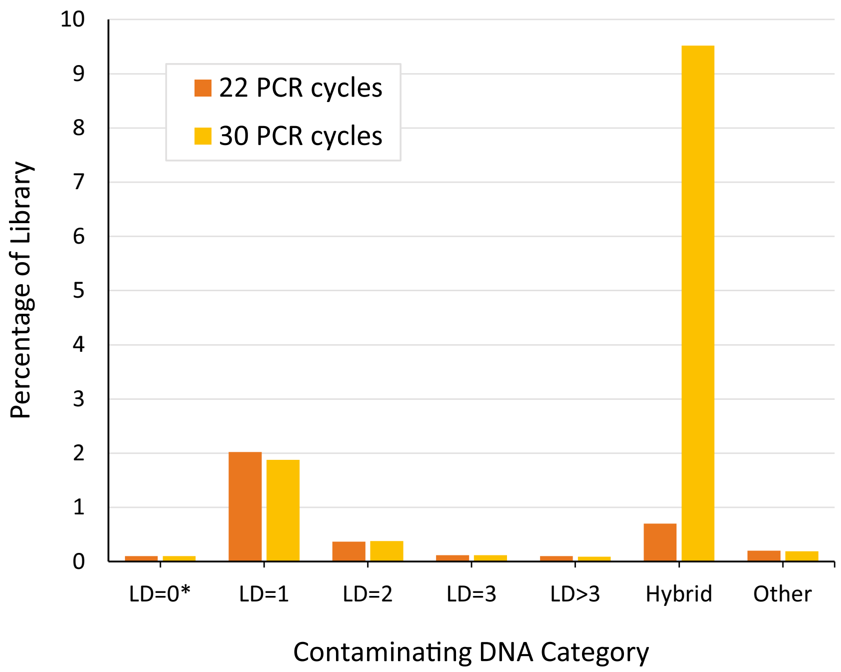

Figure EV2. The effect of PCR conditions on contaminating DNA.
Bar charts illustrating the abundance of various contaminating sequences found in the REDI molecular probe library following amplification with 22 or 30 cycles of PCR (see legend). Percent of the library is plotted on the *y*-axis for different categories of contaminating sequences (arranged on the *x*-axis). LD is Levenshtein distances of 1, 2, 3, or > 3 of a designed probes. LD = 0* represent sequences perfectly matching any of the 3,735 designed probes not targeted for cherry-picking. Hybrid probes are sequences containing 30-nt homers from two different probes. All other sequences are represented by "Other".

© 2017 The Authors

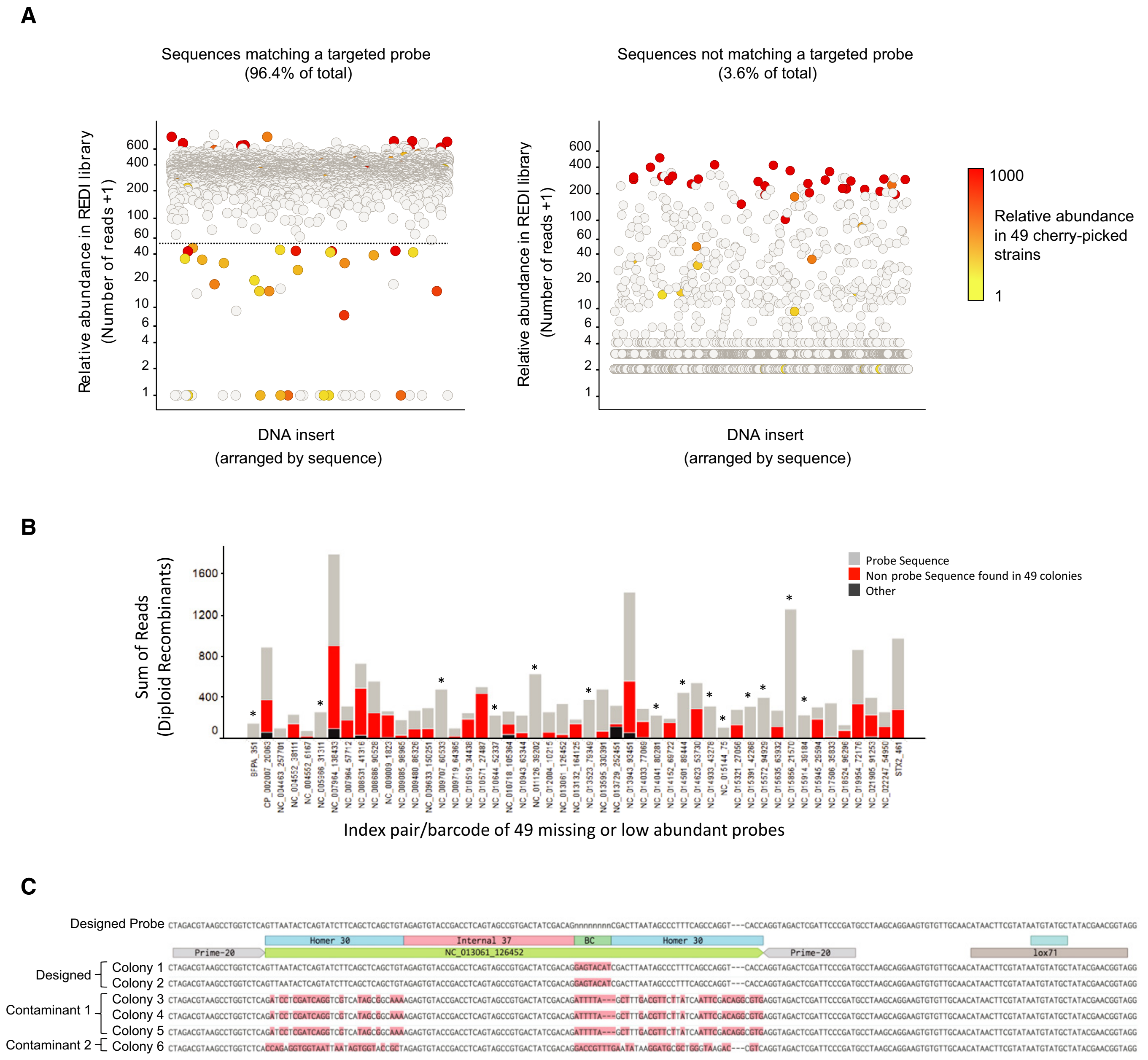

Figure EV3. Analysis of missing and contaminating DNA probes in the REDI library.

A The relative abundance (*y*-axis) of all DNA sequences found in the REDI molecular probe library. The 3,316 sequences targeted for cherry-picking are shown on the left, and all non-matching (i.e. contaminating DNA) sequences on the right. DNA is arranged alphabetically by sequence on the *x*-axis. Forty-nine sequences that are absent, or poorly represented, in the library are found below to dashed line on the left plot. The 49 colonies related to these sequences were cherry-picked from the collection, combined, and DNA inserts were amplified collectively and then analyzed by Illumina sequencing. In the plot, points are colored according to the number of sequencing reads observed in this analysis (see legend). The results reveal that these 49 colonies were the source of many of the highly abundant contaminating sequences in the REDI library (red-colored points in the right plot).

B Re-analysis of the diploid recombinant data. Bar plot illustrating the sum number of exogenous DNA reads (*y*-axis) in each of the 49 diploid recombinant colonies associated with low-abundance probes (arranged on *x*-axis by probe ID). Bars are colored according to the sequence identity (see legend). Gray represents the intended probe sequence. Secondary sequences (not matching the intended probe) and identified by > 50 reads in the sequence analysis of the 49 colonies described in (A) are colored red. All other secondary, non-matching sequences are colored black. Secondary sequences were prominently found in all but 14 of the 49 diploid colonies (asterisks above bars), indicating that in most cases, non-clonal colonies explain the poor representation of these probes.

C Analysis of a mixed colony. We selected one of the 49 strains representing poorly abundant probes, and struck it out for single colonies. From these, we randomly selected six colonies, extracted DNA, PCR-amplified the REDI locus, and Sanger-sequenced the product. Sequences were aligned to the designed probe using Benchling.

© 2017 The Authors

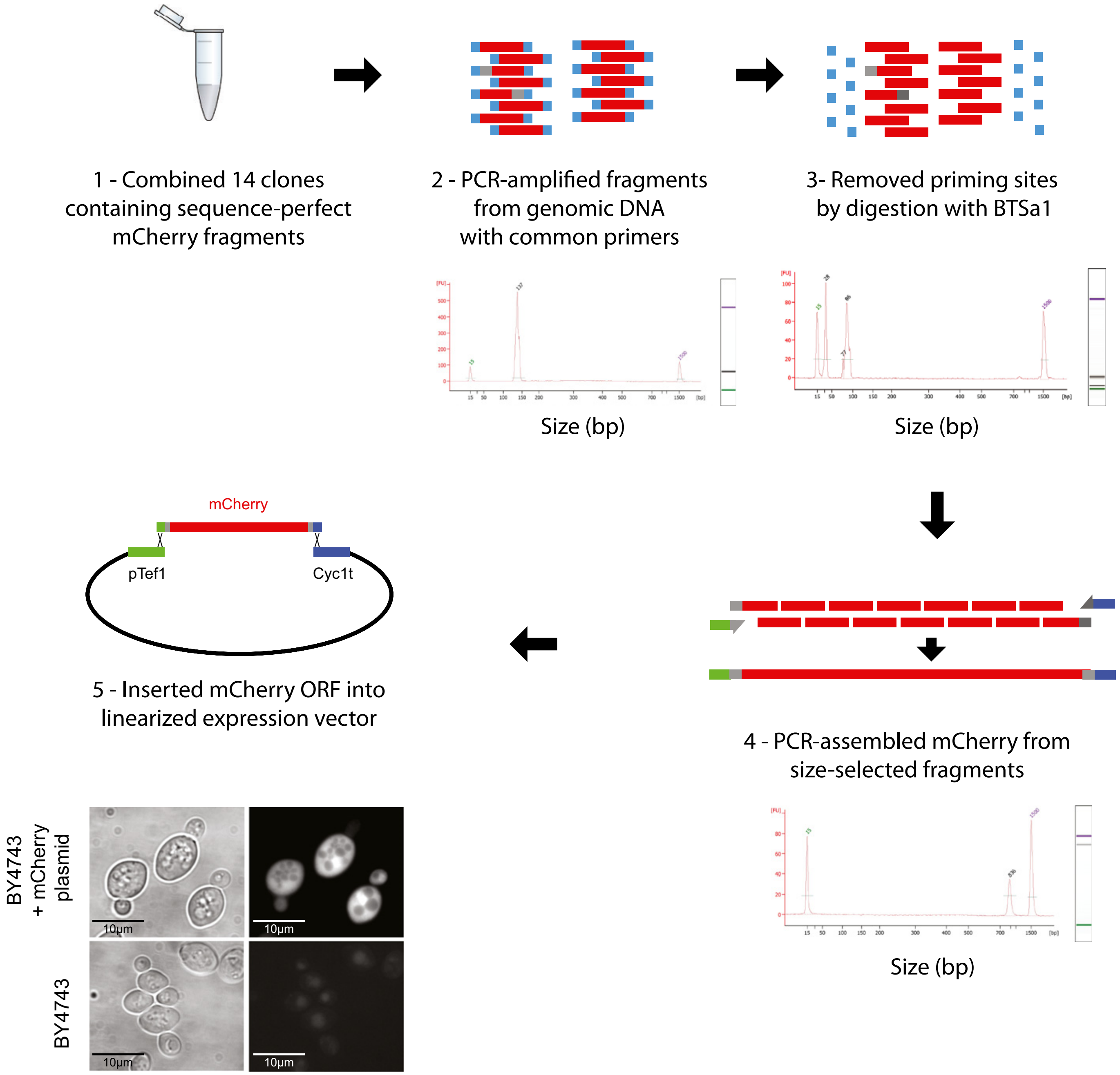

Figure EV4. Assembly of the mCherry gene from sequence-verified fragments.
(1) First, 14 yeast clones containing sequence-perfect fragments for assembly of mCherry were combined at equal concentrations and genomic DNA was isolated from this mixture. (2) From this genomic DNA, the 14 fragments were PCR-amplified using common priming sites. Below each section, a bioanalyzer trace shows that the fragments were all of the appropriate size. (3) After PCR, the common priming sites were removed by digestion with BTSa1. (4) The digested fragments were then PCR-assembled with primers that added homologies for vector integration, and size-selected to obtain fragments of the appropriate size. (5) The assembled mCherry fragment was then inserted into a linearized expression vector by yeast homologous recombination. Expression of mCherry was confirmed by microscopy. Phase contrast (left) and mCherry (right) images for BY4743 cells and BY4743 cells carrying the mCherry plasmid are shown. Sanger sequencing confirmed perfect DNA sequence in six out of six clones.

 © 2017 The Authors

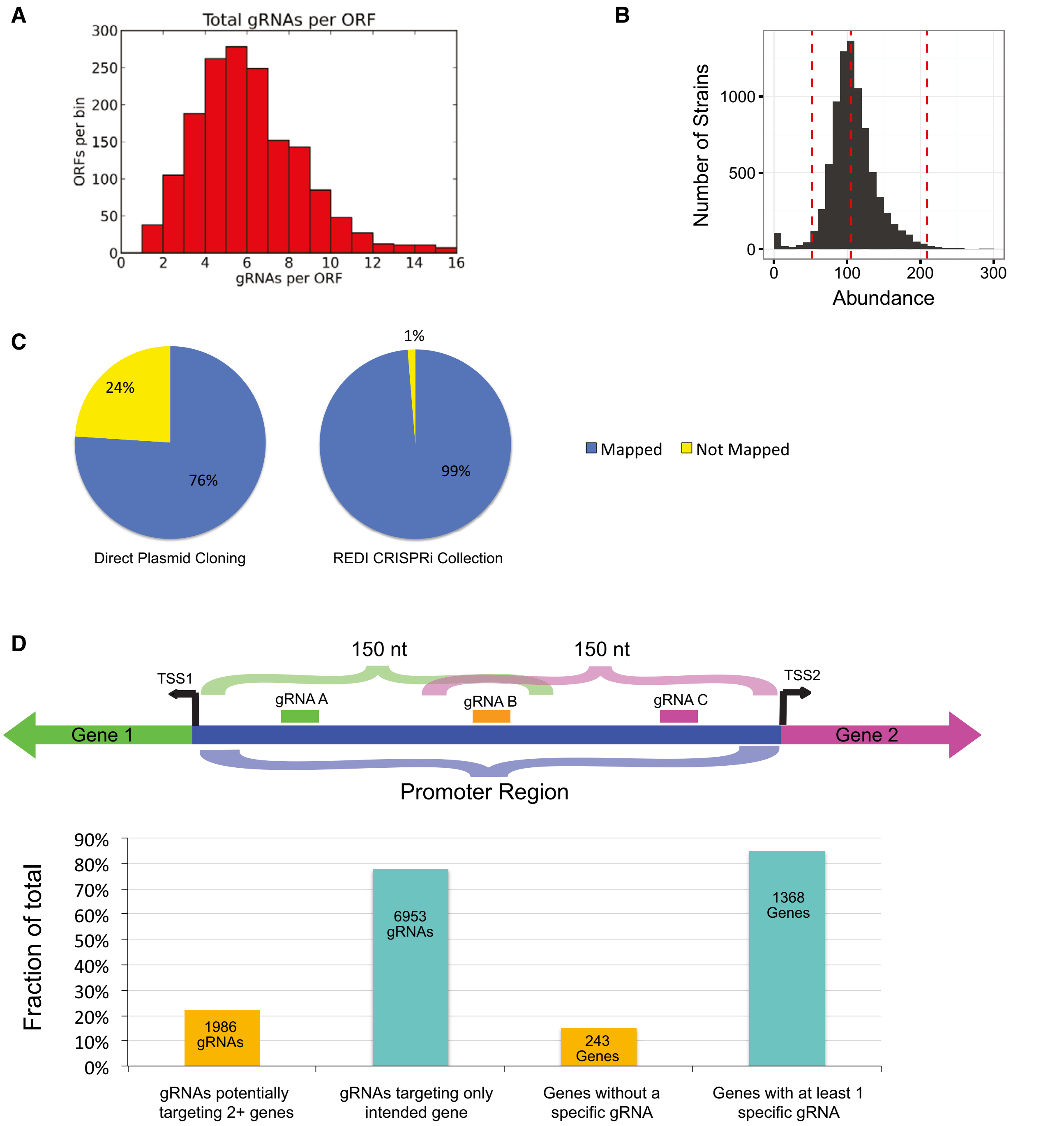

Figure EV5. The CRISPRi REDI library.

- A Histogram illustrating the number of gRNAs per ORF in the collection of 9,059 selected strains.
- B Histogram illustrating the raw sequence counts following YPD growth in the absence of ATc; 8,337 strains from "pool 1" (see Materials and Methods) are plotted. Red dotted lines demarcate 0.5×, 1×, and 2× the median.
- C Comparison of the percentage of sequencing reads mapping to designed gRNAs following competitive growth experiments using the "broad tiling library" from Smith *et al* (2016), which was directly cloned into a plasmid from a custom array oligo library (left), and using the pooled REDI CRISPRi collection (right). Reads matching a gRNA are shown in blue, and all others are shown in yellow.
- D The number of gRNAs that may repress multiple genes due to their close proximity. Guides within a window of 150 nt of the major TSS of multiple distinct ORFs (e.g. gRNA B in the schematic above) were considered to potentially target more than one ORF. The bar chart illustrates the number of gRNAs potentially targeting more than one ORF, and the number of genes without a specific gRNA based on this criterion. In the schematic, both Gene 1 and Gene 2 have at least one specific gRNA (gRNA A and gRNA C, respectively). Analyses were restricted to 8,939 gRNAs supported by at least one sequencing read, and those not targeting a dubious ORF.

© 2017 The Authors

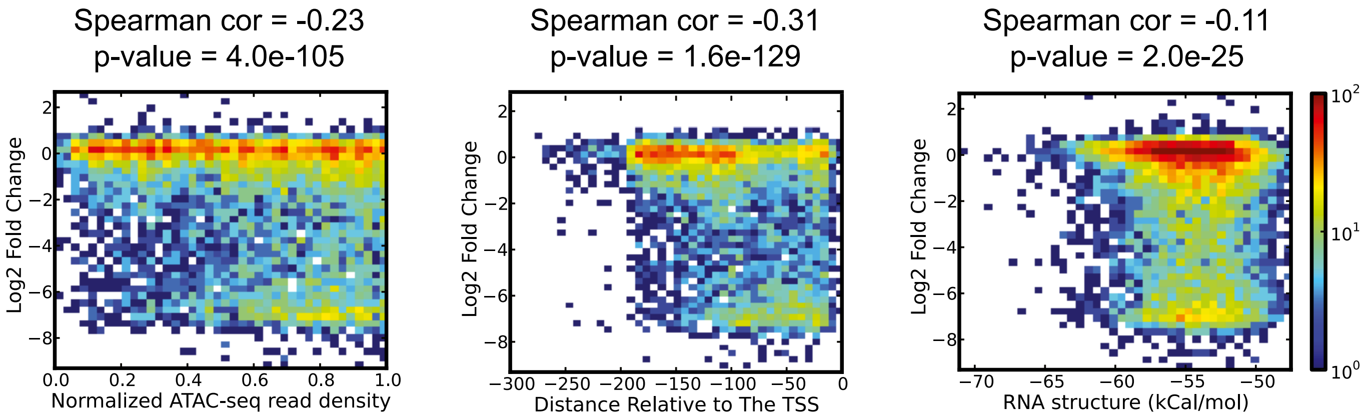

Figure EV6. Factors influencing gRNA efficacy.

Density heat maps, where colors show relative number of gRNAs per bin (see legend). CRISPRi-induced growth defects (*y*-axes) are plotted against three gRNA parameters (from left to right: chromatin accessibility, position relative to the TSS, and secondary structure).

Figure EV7. Effect of *IRA1* or *IRA2* repression on respiratory growth.

A Optical density (*y*-axis) was measured every ~15 min for ~62.5 h (*x*-axis) in 96-well microtiter plates. Each panel plots six technical replicates grown in the presence (red lines) or absence (black lines) of 250 ng/ml ATc. Six *IRA1* and *IRA2* repressor strains and a control strain (Ctrl gRNA) expressing a gRNA with no homology to the yeast genome are indicated above. Results for growth assays performed on three different days are arranged in each row and labeled by date on the far right.

B As in (A), only the increase in growth observed in each well after cells have entered stationary phase (45–55 h following the start of the experiment) is plotted.

C Relative growth, calculated from OD measurements collected during the first 30 h of growth, is plotted for individual biological replicates (see legend).

D As is (C), only relative growth was calculated after cells have entered stationary phase (45–55 h following the start of the experiment).

© 2017 The Authors

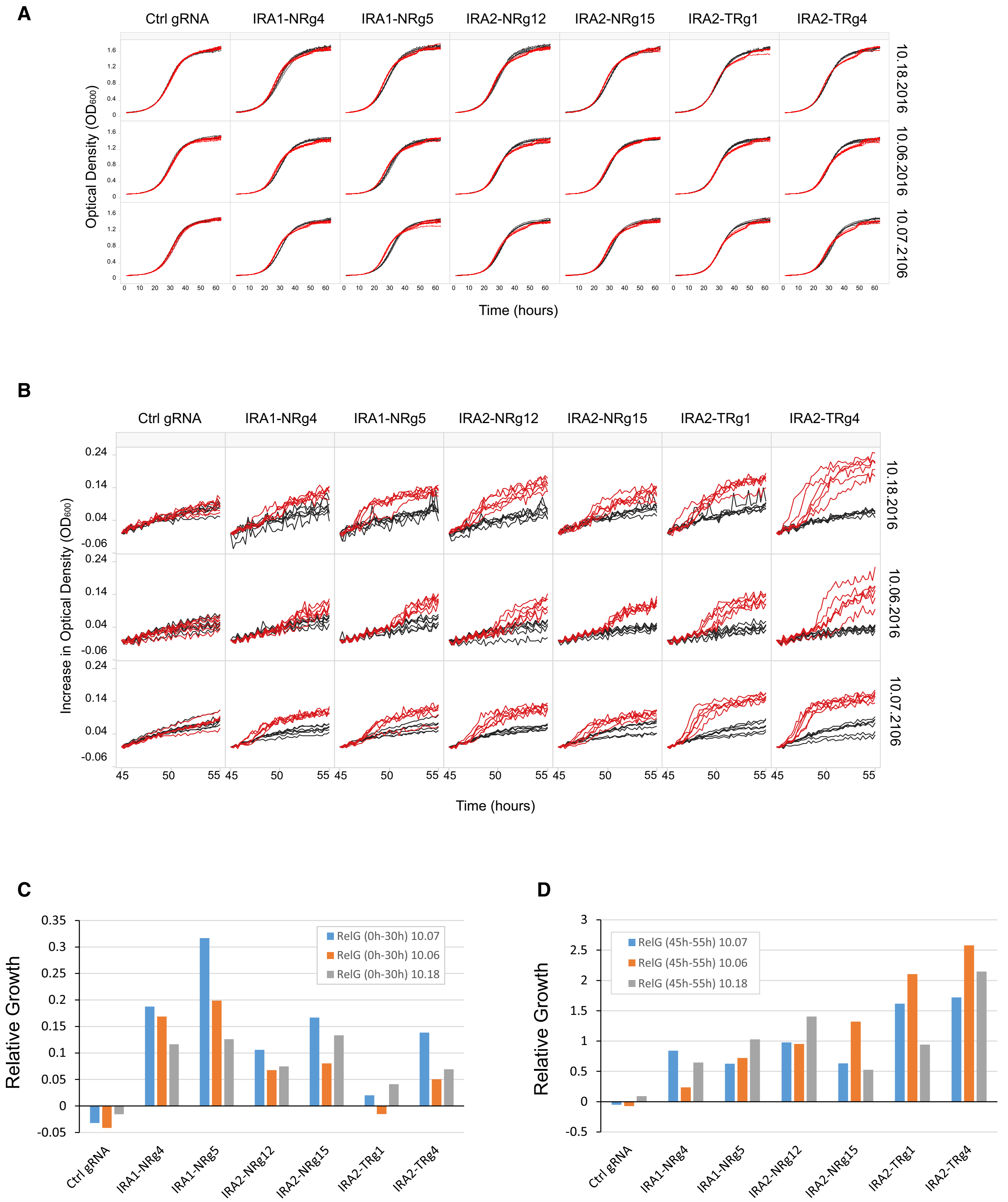
A
Ctrl gRNA
IRA1-NRg4
IRA1-NRg5
IRA2-NRg12
IRA2-NRg15
IRA2-TRg1
IRA2-TRg4
Optical Density (OD600)
10.18.2016
10.06.2016
10.07.2106
Time (hours)
B
Increase in Optical Density (OD600)
Time (hours)
C
Relative Growth
RelG (0h-30h) 10.07
RelG (0h-30h) 10.06
RelG (0h-30h) 10.18
D
RelG (45h-55h) 10.07
RelG (45h-55h) 10.06
RelG (45h-55h) 10.18

Figure EV7.
